# Prospective prediction of alcohol consumption among a Tunisian sample of adolescents

**DOI:** 10.1093/eurpub/ckac131.462

**Published:** 2022-10-25

**Authors:** N Zammit, H Ghali, R Ghammam, S Ben Fredj, W Ben Belgacem, S Boujebha, M Ouertani, A Maatouk, J Maatoug, H Ghannem

**Affiliations:** 1 Department of Epidemiology, University Hospital Farhat Hached, Sousse, Tunisia; 2 Faculty of Medicine of Sousse, University of Sousse, Sousse, Tunisia; 3 LR19SP03, University Hospital Farhat Hached, Sousse, Tunisia; 4 Department of Prevention and Security of Care, University Hospital Sahloul, Sousse, Tunisia; 5 LR20SP06, University Hospital Sahloul, Sousse, Tunisia

## Abstract

**Background:**

During adolescence, alcohol consumption represents a new experience with the advantage of facilitating the integration of a peer group. The global overall prevalence of this risk behavior among the 15-19 years old was over 25% in 2018. However, this prevalence varies between countries.

**Objectives:**

To determine the incidence and the predictors of alcohol consumption among high school students in Sousse, Tunisia between 2017/2018 and 2018/2019.

**Methods:**

A prospective longitudinal study was conducted in four high schools in the governorate of Sousse during the 2018-2019 school year. Pre-trained medical doctors used an anonymous self-administered questionnaire to collect data about socio-demographic and educational features, alcohol consumption, tobacco use, illicit substances use and emotional disorders.

**Results:**

Participants accounted for 404. Their average age was 16.4 (±1.1) years. Females represented 68%. The prevalence of alcohol consumption in 2017/2018 was 5.9% while the cumulative incidence during the 2018/2019 school year was 3.5%. Among males, this incidence was of 11.9%. Among females, it was of 3% (p = 0.020). Illicit substance use among friends was the main predictor of becoming alcohol consumer with an adjusted odds ratio of 6.4 (95% CI: 1.9-21.3) on the other hand, having an anxiety trouble predicted less this risk behavior (adjusted odds ratio =0.2 95% CI: 0.1-0.8).

**Conclusions:**

Alcohol consumption is becoming more and more common among the adolescents of Sousse especially among males. The current national strategy against substances use in schools should be reinforced. Implementation of a social skills training among adolescents to improve assertiveness is essential.

**Key messages:**

• Alcohol consumption has an upword trend among the adolescents of Sousse.

• The current prevention programs targetting adolescents in Tunisia should be revised and integrate a comprehensive and multisectoral program.

